# Indications Selection for Surgeons Training in the Translaminar Percutaneous Endoscopic Discectomy Based on Finite Element Analysis

**DOI:** 10.1155/2020/2960642

**Published:** 2020-02-07

**Authors:** Jingchi Li, Wenqiang Xu, Qingfeng Jiang, Zhipeng Xi, Xiaoyu Zhang, Nan Wang, Lin Xie, Yang Liu

**Affiliations:** ^1^Department of Spine Surgery, Affiliated Hospital of Integrated Traditional Chinese and Western Medicine for Nanjing University of Chinese Medicine, Nanjing 210028, Jiangsu, China; ^2^Department of Anatomy, School of Basic Medical Sciences of Southwest Medical University, Luzhou 646000, Sichuan, China; ^3^Department of Spine Surgery, Changzheng Hospital Affiliated to the Naval Medical University, Shanghai 200041, China

## Abstract

**Background:**

Translaminar percutaneous endoscopic discectomy (PED) was used widely in the treatment of lumbar disc herniation (LDH), especially for the training of novice surgeons. A larger range of osteotomy was a suitable method to get enough operation space and reduce intraoperative risks. But osteotomy, especially facetectomy, may be associated with the biomechanical deterioration and resulting adjacent segment diseases (ASD). Hence, the objects of this study were to investigate whether different levels of surgical experience in performing different ranges of osteotomy (especially facetectomy) affected the risk for ASD and to identify the safe indications for the training of PED novice surgeons. *Study Design*. In this study, a three-dimensional lumbosacral model was constructed and validated. Corresponding translaminar PED models with different ranges of osteotomy for armpit, periradicular, and shoulder types of LDH were constructed. The von Mises stress on the endplates, the shear stress on the annulus, the intradiscal pressure, and the range of motion (ROM) in the L3-L4 segment disc were computed.

**Results:**

Computational results in our well-validated model indicated that large ranges of osteotomy led to deterioration in most of the biomechanical indicators, and this trend was most significant in the shoulder-type LDH model.

**Conclusions:**

To ensure the appropriateness of the surgical prognosis, armpit and periradicular types of LDH can be seen as suitable indications for the training of novice PED surgeons, and shoulder-type LDH should be excluded from such indications until novices can perform PED within a relatively small range of osteotomy. *Mini Abstract*. Based on biomechanical variations in our finite element analysis, armpit and periradicular types of LDH can be seen as suitable indications for the training of novice PED surgeons, and shoulder-type LDH should be excluded until novices can perform PED within a relatively small range of osteotomy.

## 1. Introduction

Percutaneous endoscopic discectomy (PED) has been used extensively in the treatment of lumbar disc herniation (LDH) in recent years [1]. The translaminar approach for PED, which is similar to the open posterior lumbar surgery in the anatomic characteristics, is promoted in the training of novice PED surgeons.

In comparison with traditional open posterior surgery, PED should decrease the risk of adjacent segment disease (ASD) which was partly caused by a wide range of postoperative paraspinal muscle atrophy and epidural adhesion [2–4]. According to the literature, PED could nonetheless lead to biomechanical deterioration, the most important factor in the development of ASD [5–9].

Osteotomy, such as facetectomy, has been proven to be associated with ASD. For instance, in 15%–37% of cases of low back pain, a typical symptom of ASD, the cause is related to injury of facet joints [1, 10, 11]. The facet cartilage and joint capsules play key roles in protecting the posterior annulus [12, 13] and in maintaining lumbar stability [5, 7, 14]. Iatrogenic instability and annulus tears are key triggers of ASD [7, 15, 16], which may lead to low back pain through the increased stimulation of localized nociceptors [17, 18].

**Figure 1:**
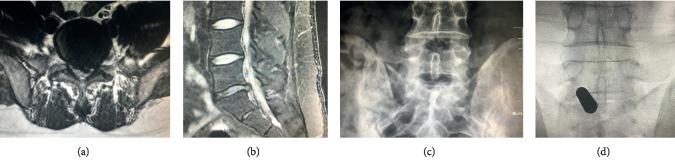
The imaging findings of typical cases of osteotomy in PED. (a) MRI in the coronal plane. (b) MRI in the sagittal plane. (c) Prospective X-ray scan. (d) Intraoperative X-ray scan. The patient in the figure was a 38-year-old man whose height was 180 cm. The herniated nucleus could be identified on the coronal plane of MRI from the medial to the lateral side of the nerve root. Preoperative anterior and posterior X-ray images revealed the width of the interlaminar space, whereas intraoperative X-ray using C-arm fluoroscopy revealed the diameter of the working tube to be considerably larger than that of the interlaminar space.

**Figure 2:**
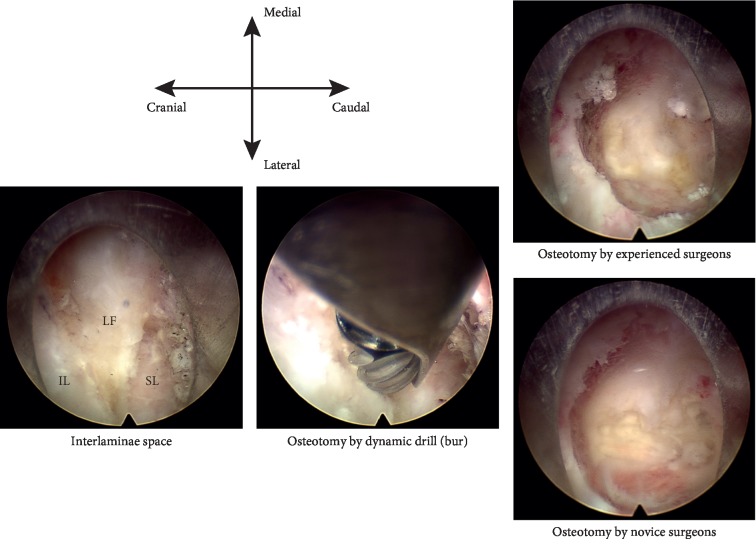
The intraoperative endoscopic images. LF : ligamentum flavum; IL : inferior laminae; SL : superior laminae.

But osteotomy is essential in translaminar PED for patients with narrow interlaminar spaces and shoulder-type LDH. For these patients, the anatomic osseous diameter of the interlaminar space does not allow the insertion of a working tube into the spinal canal through the ligamentum flavum. As a result, the osteotomy is essential in the surgical procedure, and limited osteotomy hinders the insertion of the working tube and the traction on the nerve root (Figures 1 and 2) [19–23].

As a result, it increases the incidence of intraoperative discomfort in patients not receiving general anesthesia, as well as the risk for nerve root injury and inadequate decompression. Hence, a larger range of osteotomy may be necessary, especially in PED performed by novice surgeons. In contrast, surgeons with experience in PED can achieve the surgical purpose in a relatively small space and the range of facetectomy can also be reduced.

On the basis of these theories and practical observations, we hypothesized that larger ranges of osteotomy, especially facetectomy, in translaminar PED performed by novice surgeons may increase the risk for ASD; such a trend should be reflected by variations in postoperative biomechanical indicators. To the best of our knowledge, this issue has not been clarified adequately in literature published to date. To verify this hypothesis and provide theoretical guidance about training novice PED surgeons, we constructed and validated a lumbosacral finite element analysis (FEA) model and corresponding PED models for the armpit, periradicular, and shoulder types of LDH. Different ranges of osteotomy were used to simulate this operation by novice and experienced surgeons.

## 2. Material and Methods

### 2.1. Model Construction

FEA models from L3 to S1 were constructed by standard curves. Bone structures include cortical, cancellous, and posterior structures reconstructed in our previous studies which were used as the templates for model construction [8, 9, 24]. Different parts of the templates were layered, and the outline in each layer was traced by standard curves.

Nonbony components include intervertebral discs, facet joints, and ligaments. Intervertebral discs consist of the inner nucleus, the surrounding annulus, and cranial and caudal endplates. The contours of nonbony components, like the bone structures, were constructed according to standard curves. The average radius of annuli and endplates was set as 95.5% of values of corresponding to the vertebral body, the cross-sectional areas of the nucleus were confirmed as 38% of intervertebral discs, and the ratio of the distance between the anterior edge of the annulus and the nucleus to the distance between the posterior edge of the annulus to the nucleus was set as 1.62 to define its relative position.

The thickness of the cortical and the endplates was set at 0.8 mm, and the gap between the facets was set as 0.5 mm [25]. The anterior and posterior longitudinal ligaments, the ligamentum flavum, the intertransverse ligament, the interspinous and supraspinous ligaments, and the joint capsules of the models were constructed in the FEA preprocessing phase by tension-only cable elements [9, 25].

### 2.2. Simulation of Translaminar Percutaneous Endoscopic Discectomy

The simulation of PED was based on the published literature and our clinical experience [21, 23]. PED models were constructed on the right side of the L4-L5 segment and were divided into the armpit, periradicular, and shoulder types of LDH. Three 4 mm incisions on the annulus were made in models with different types of LDH, respectively; one in the medial border of the pedicle; one in the posterior midline and another one in the midline between the other two. To represent discectomy, one-third of the nucleus was deleted in corresponding regions [8, 9].

**Figure 3:**
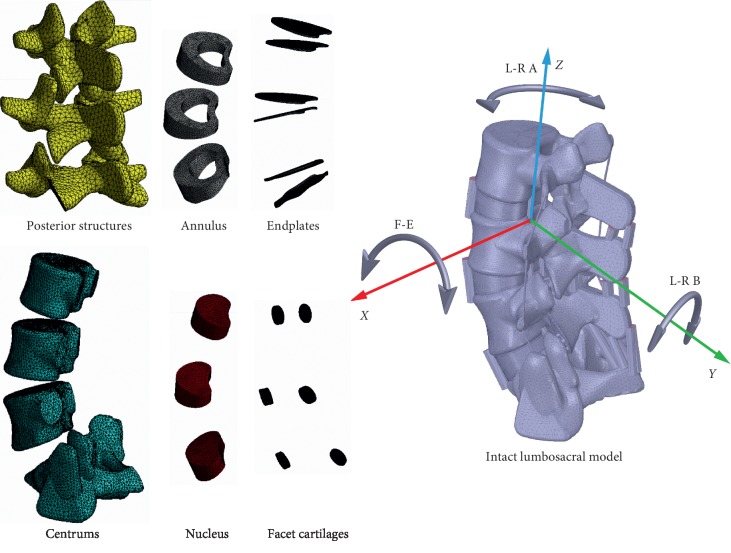
The intact FEA model in this study.

**Figure 4:**
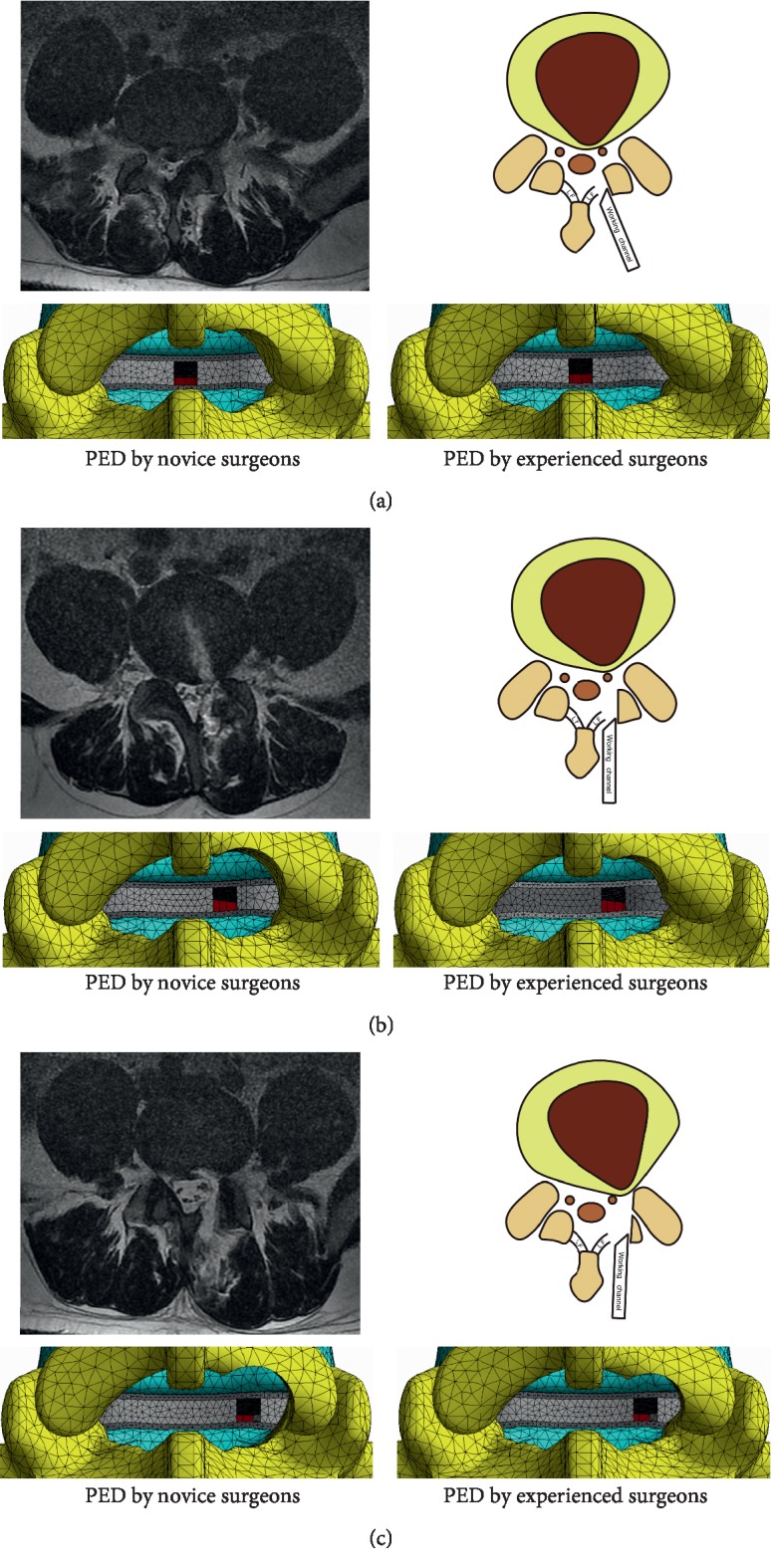
The simulation of PED by beginners and experienced surgeons. (a) Armpit-type LDH. (b) Periradicular-type LDH. (c) Shoulder-type LDH.

To simulate the osteotomy by novice and experienced PED surgeons, 13.5 and 11.5 mm diameter circles around the central point of annulus incision were drawn, and osteotomies of the laminar and facet joints were outlined. The diameter of the endoscopic working tube in our department was 7.5 mm (type WTS127500, Joimax International, Irvine, Calif.), and 3 and 2 mm enlargement was defined in all directions to simulate the needed operation spaces for novice and experienced surgeons. Facet cartilages and joint capsules within the range of osteotomy were excised together. In addition, one-third of the ligamentum flavum on the surgical side for armpit and periradicular types and quarter of which for shoulder-type LDH was removed. The schematic of the intact model is shown in Figure 3, and the PED models are shown in Figure 4.

### 2.3. Boundary and Loading Conditions

Models were constructed to be symmetric in the sagittal plane. Different sizes of tetrahedron elements were used in the mesh generation, and the mesh was refined in thin structures and the structures with large deformation. The average mesh quality was greater than 0.75 in current models and greater than 0.8 in structures with mesh refinement. Bounded contact type was used for all surfaces except for facet cartilages, in which contact was defined as frictionless [9, 26]. All degrees of freedom were fixed below S1, and stress was applied superior to L3 [25, 26].

To validate whether current FEA models are adequate representations of real situations in different body positions, the values for range of motion (ROM) in the surgical segment (L4-L5) were computed and compared with those from widely cited *in vitro* studies under same loading conditions (100 N vertical compression with 10 N·m moments in different directions). Biomechanical indicators used to evaluate the risk of ASD were computed under different directions of 10 N·m moments without vertical compression [26–28]. What we needed to illustrate was that the intact model was strictly symmetric and placed symmetrically along the sagittal plane, so that lateral bending and axial rotation were computed only in the rightward direction. However, PED models were not symmetric; therefore, they were computed in both leftward and rightward directions. The material properties of the models are listed in Table 1.

## 3. Results

### 3.1. Model Validation

**Figure 5:**
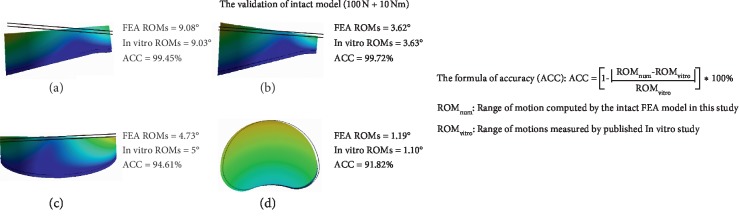
Validation process of the current FEA model. (a) Flexion. (b) Extension. (c) Bending. (d) Rotation.

The concept of accuracy (ACC) came up as an indicator for model validation and ACC is greater than 90% under all loading conditions (Figure 5) [29]. This result illustrates that our models were good representations of real situations in different body positions and could be used in this study.

### 3.2. The Evaluation of Risk for ASD

The L3-L4 segment was selected for the evaluation of ASD risk. The maximum von Mises stress on the endplates, the maximum shear stress on the annulus, the intradiscal pressure, and the ROM were computed to evaluate the variation in ASD risk. Although the variations of extents in different biomechanical indicators were slightly different, their general tendency was consistent in our FEA study.

**Figure 6:**
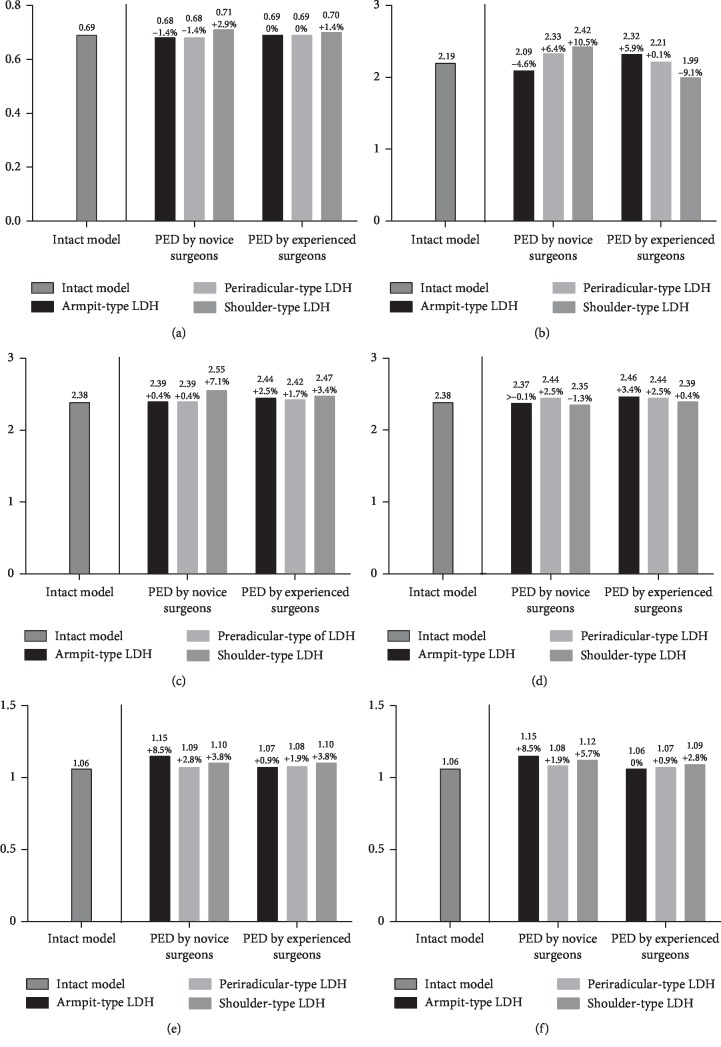
Histograms of variation of endplates maximum von Mises stress. (a) Flexion. (b) Extension. (c) Left lateral bending. (d) Right lateral bending. (e) Left axial rotation. (f) Right axial rotation.

**Figure 7:**
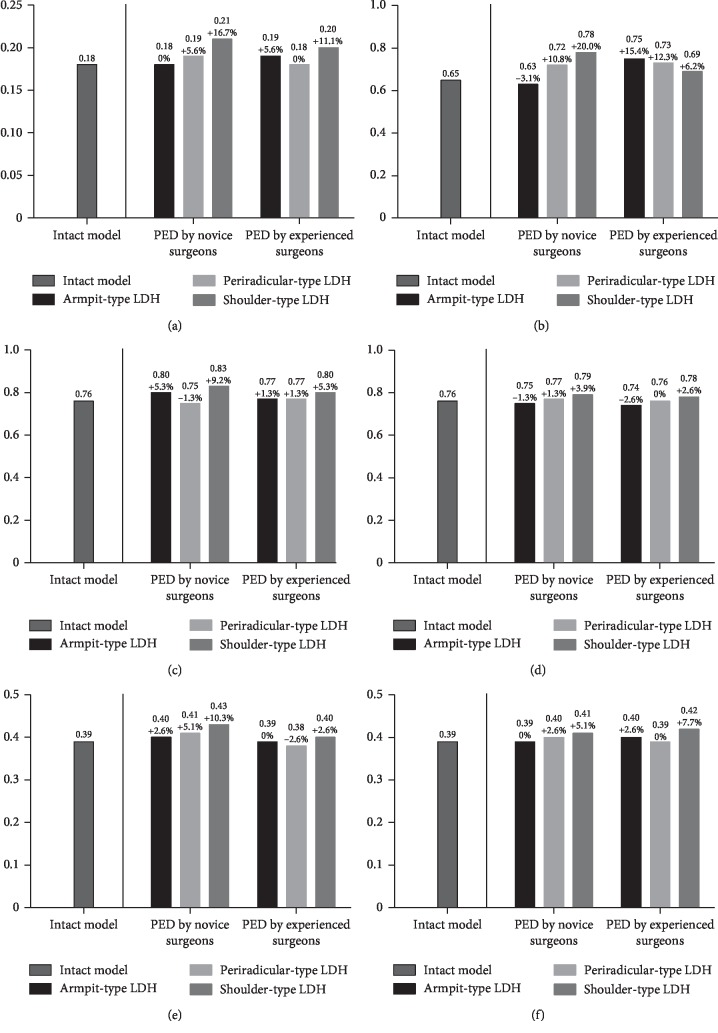
Histograms of variation of annulus maximum shear stress. (a) Flexion. (b) Extension. (c) Left lateral bending. (d) Right lateral bending. (e) Left axial rotation. (f) Right axial rotation.

**Figure 8:**
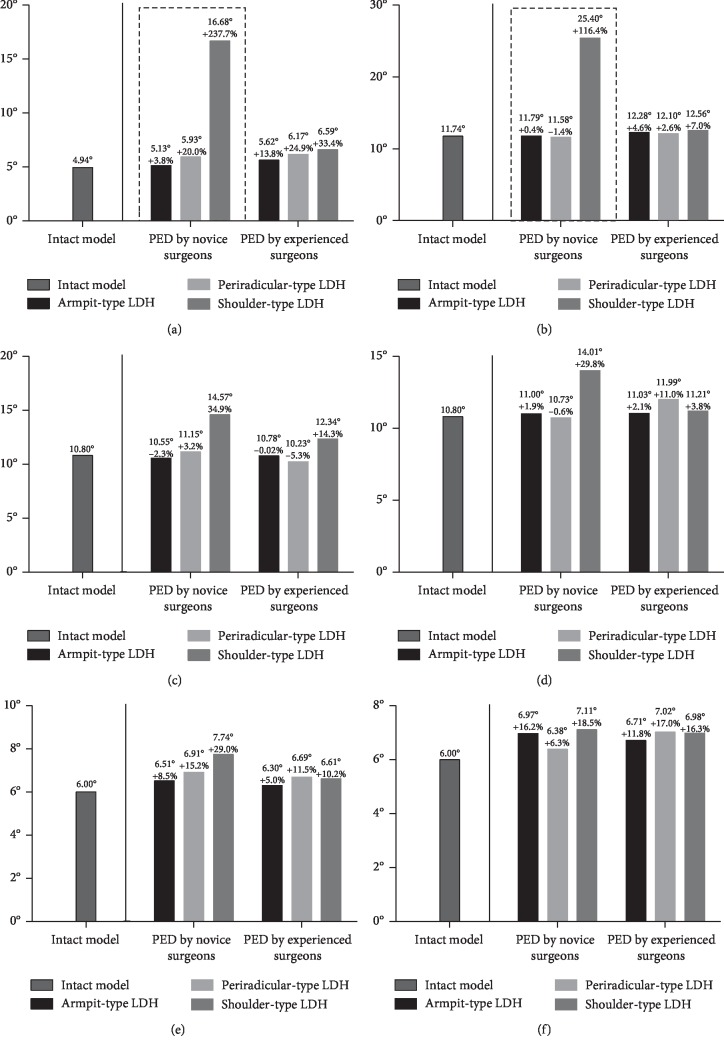
Histograms of variation of ROM. (a) Flexion. (b) Extension. (c) Left lateral bending. (d) Right lateral bending. (e) Left axial rotation. (f) Right axial rotation.

**Figure 9:**
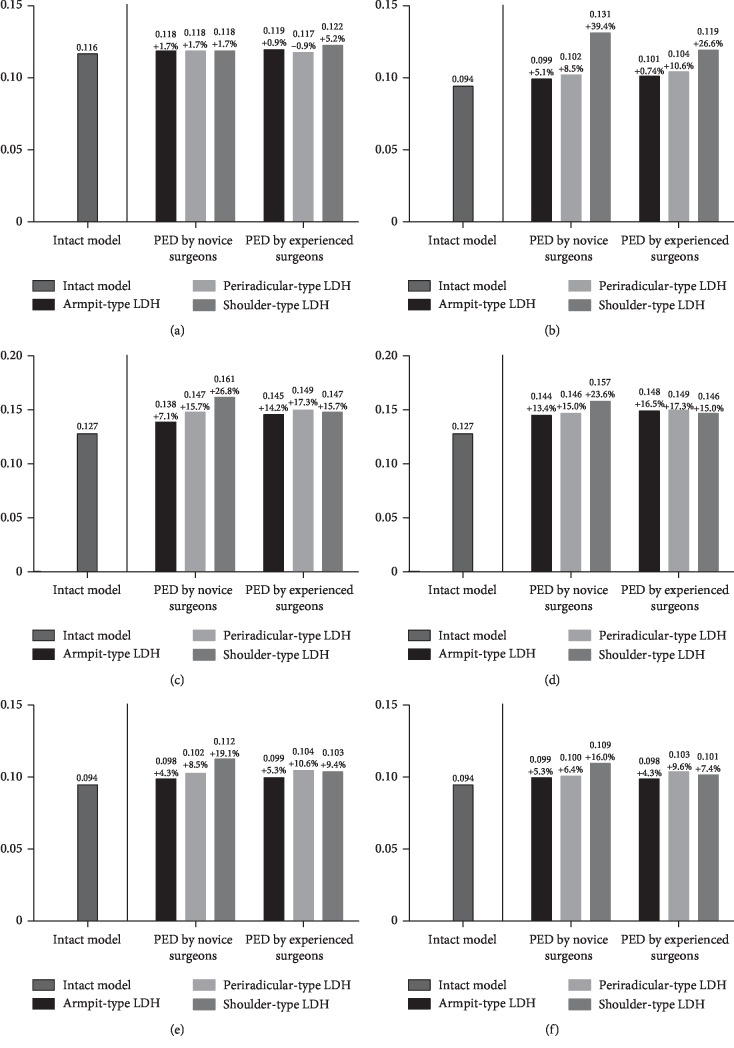
Histograms of variation of intradiscal pressure. (a) Flexion. (b) Extension. (c) Left lateral bending. (d) Right lateral bending. (e) Left axial rotation. (f) Right axial rotation.

**Figure 10:**
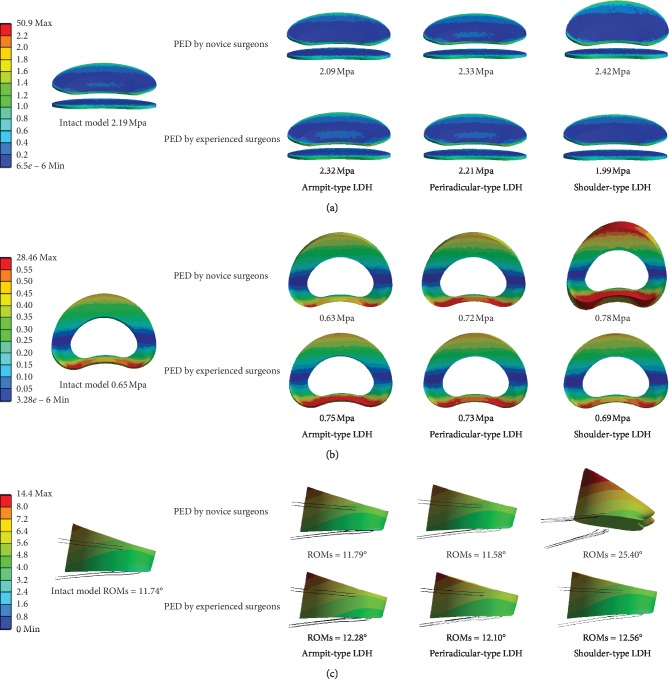
Nephrograms of biomechanical variations under extension condition. (a) The nephrograms of endplates von Mises stress (MPa). (b) The nephrograms of annulus shear stress (MPa). (c) The nephrograms of ROMs.

In general, the values of maximum stress, intradiscal pressure, and ROM were increased in PED models compared with values from the intact model. And the deterioration of biomechanical indicators was more obvious in the models of simulated surgery by novice PED surgeons than which by experienced surgeons. The variation in von Mises stress in the endplates was slight, and in ROM was most evident. The trend of biomechanical deterioration was most dramatic in the shoulder-type LDH model after PED by novice surgeons, in which the areas of shear stress concentration and ROM increased obviously. Histograms and nephrograms of different biomechanical indicators are shown in Figures 5–10.

## 4. Discussion

The inspiration for this study was the intraoperative confusion faced by novice PED surgeons in our department. Unskilled surgical operations by novices in limited operating spaces cause intraoperative discomfort in patients not receiving general anesthesia; this in turn may cause patients to cry out in pain and complain, which may increase tension for inexperienced surgeons and lead to increases in nerve root injury and inadequate decompression, the two most important reasons for poor postoperative short-term outcome [30–33]. Hence, for novice PED surgeons, a larger range of osteotomy was a suitable method to get enough operation space and reduce the above risks. And in this study, the variations of biomechanical indicators in adjacent segments caused by different ranges and positions of osteotomy in translaminar PED were computed to investigate whether training of novice PED surgeons would increase the risk of ASD for different types of LDH, and current findings are of significance for the implications selection in the training of novices.

Additionally, as Figures 1 and 2 illustrate, the patient described in the present report was 180 cm tall with no obvious narrowing of the intervertebral space observed on preoperative sagittal magnetic resonance imaging (MRI). Nevertheless, the interlaminar space could not accommodate to the working tube required for osteotomy. In this situation, discectomy cannot be performed without the use of a dynamic drill (bur). It is well known that, on average, Asian patients are shorter than Caucasian patients. As a result, the interlaminar spaces of Asian patients will be narrower than that of Caucasian patients; therefore, more cases may require osteotomy and facetectomy in PED. Additionally, endoscopic images presented in the right side of Figure 2 illustrate the differences in osteotomies carried out by novice and experienced surgeons, clearly demonstrating that experienced surgeons can perform this surgery in a relatively smaller space and that a larger range of osteotomy is required by novice surgeons.

We did not simulate PED and evaluate the risk of ASD in the L5-S1 segment because the inferior surfaces of S1 were fixed and the resulting values of ROM would have been lower in this segment. Comparison of the ROM in the FEA model with values from widely cited *in vitro* studies is a standard model validation procedure of FEA study, and the variation in ROM was an important indicator of ASD [8, 9, 26, 34]. The surgical simulation and ASD risk evaluation in this segment would have decreased the accuracy and credibility of our study. In addition, published literature has illustrated that ASD was more common in the cranial rather than in the caudal segments [35, 36]. The simulation of PED in the L4-L5 segment allowed us to analyze the variation in ASD risk at the L3-L4 segment, a segment in which natural disc degenerative changes are far less common than in L4-L5 and L5-S1 segments, and get a more credible conclusion.

Although degeneration in the surgical segment originally proved to increase the risk of ASD [7, 24], our findings still verify our hypothesis in PED models without the presence of degeneration. The reason was that the construction of the degenerative model accounted for changes in the disc height, the cross-sectional area of the nucleus, the material properties of the disc, and the gap between the facets of the model without degeneration [6, 24]. This approach does not account for the hypertrophy of the articular process, the cohesion, and consequent spinal stenosis and nerve compression in the degenerative segment. In other words, a larger range of osteotomy in the degenerated segment was intended not only just for the purposes mentioned earlier but also for nerve decompression directly. In addition, the gradual mastery of increasingly complex operations is a general rule among inexperienced surgeons, and complicated situations caused by a degenerative change in the surgical segment will cause the surgery to be more difficult. In other words, training of novice PED surgeons should begin with cases that are mastered easily: specifically, in cases without degenerative changes. Thus, FEA study without degeneration models could still achieve our research objective.

A change of motility in a particular segment causes pathological compensation in adjacent segments [5] and increases the risk of lumbar instability, which can lead to ASD and be reflected by the increase in ROM [7, 14–16]. The largest ROM could observed in the shoulder-type LDH model after PED by novice surgeons with the highest ranges of facetectomy, which can be seen as an important basis of lumbar instability and resulting ASD and is consistent with the theory that the articular process is essential for maintaining lumbar stability [5, 14, 37].

Meanwhile, endplates play a key role in the pressure distribution. The concentration of stress on endplates increases the risk for microfractures under the endplates, which impede the trans-endplate nutrition diffusion, which is the most important pathway of the metabolism of adult discs [38, 39], accelerates disc degeneration, and automatically increases the risk for ASD [5, 40]. Moreover, endplate injury is closely associated with the disruption of the annulus, and the increase in maximum shear stress and areas of shear stress concentration in the posterior annulus has been regarded as an important contributor to annulus tears [5, 24]. Annulus tears, alone with the increase of intradiscal pressure, are important causes of LDH in adjacent segment disc and discogenic LBP, which can naturally be considered as triggers of ASD [5, 8, 9, 34].

Although the increase in endplates maximum von Mises stress and annulus maximum shear stress was relatively slight in the shoulder-type LDH model after PED by novice surgeons, our deduction is still reliable and predictive. Most of the indicators of biomechanical performance showed the same trends in this study and 10 N·m moments, the moment maximum ROM in which was less than 30°, were relatively small compared with the strenuous activities in our daily life and we can speculate that these changes may be amplified under cyclic loading and larger moments in actual situations. In addition, as is shown in Figure 10, the areas of annulus shear stress concentration increased obviously and slight biomechanical deterioration have been proven to be a potential cause of severe disc degeneration [26]; we believe that the above basis is sufficient to support our following conclusion.

The computational results indicated that larger ranges of osteotomy by novice PED surgeons will increase the risk of ASD in varying degrees, and this trend was dramatically obvious in shoulder-type LDH. Hence, we can make the conclusion that to ensure the appropriateness of the surgical prognosis, armpit and periradicular types of LDH can be seen as suitable indications for the training of novice PED surgeons, and shoulder-type LDH should be excluded from such indications until novices can perform PED within a relatively small range of osteotomy.

Moreover, Figure 4 shows that 1 mm enlargement of the surgical space would not obviously increase the degree of osteotomy in the armpit and periradicular types of LDH; however, this was not the case for shoulder-type LDH. In such a situation, the osteotomy enlargement would not only increase the area of facetectomy but also aggravate the damage of facet cartilage and capsule, structures important for the annulus protection and lumbar stability maintenance [5, 12, 14, 37], and increase the risk for ASD. Our conclusions are supported from this perspective as well.

There were still several limitations to this study. For instance, ligaments were constructed by cable elements, and the simulation of excision of the ligamentum flavum and the joint capsule was accomplished by changing the cross-sectional areas; this method was inaccurate and may represent a weakening of their biomechanical roles. Meanwhile, the conclusions based on our results are inferential and should be proved with larger moments and cyclic loading in future studies.

## 5. Conclusions

To ensure the appropriateness of the surgical prognosis, armpit and periradicular types of LDH can be seen as suitable indications for the training of novice PED surgeons, and shoulder-type LDH should be excluded from such indications until novices can perform PED within a relatively small range of osteotomy.

## Figures and Tables

**Table 1:** Material properties of current FEA models.

	Young's modules (MPa)		Poisson ratio	Cross-sectional areas (mm^2^)	Element types	Element sizes (mm)
Cortical	12000		0.3			2.0
Cancellous	100		0.2			3.5
Posterior structures	3500		0.25			2.8
Endplates	1000		0.4	—	Tetrahedral	0.8
Cartilages	10		0.4			0.6
Annulus	4.2		0.1			1.3
Nucleus	1		0.499			1.8
Capsules	7.5 (*a* < 25%)	33 (>25%)	0.3	30		—
ALL	8 (<12%)	21 (>12%)	0.3	60		—
PLL	11 (<11%)	22 (>11%)	0.3	21		—
LF	15 (<6.2%)	19 (>6.2%)	0.3	60	Cable	—
ITL	10 (<18%)	59 (>18%)	0.3	10		—
ISL	10 (<14%)	12 (>14%)	0.3	40		—
SSL	9 (<20%)	16 (<20%)	0.3	30		—

ALL: anterior longitudinal ligament; PLL: posterior longitudinal ligament; LF: ligamentum flavum; ITL: intertransverse ligament; ISL: interspinous ligament; SSL: supraspinous ligament (SSL).

## Data Availability

All the data of the manuscript are presented in the paper

## Abbreviations

PED: Percutaneous endoscopic discectomy
LDH: Lumbar disc herniation
ASD: Adjacent segment diseases
LBP: Low back pain
MRI: Magnetic resonance imaging.
